# Navigating the brain: How cerebral blood flow shifts with task complexity

**DOI:** 10.1371/journal.pone.0333684

**Published:** 2025-10-23

**Authors:** Laura K. Fitzgibbon-Collins, Mamiko Noguchi, Geoff B. Coombs, Richard L. Hughson, Corey Guest, Robert Amelard, Dave McArthur, Sue Peters, Sarah Best, Michael Borrie, Habib Khan, J Kevin Shoemaker, Jaspreet Bhangu

**Affiliations:** 1 Department of Medicine, Division of Geriatric Medicine, Schulich School of Medicine & Dentistry, University of Western Ontario, London, Ontario, Canada; 2 School of Kinesiology, Western University, London, Ontario, Canada; 3 Department of Kinesiology, University of Waterloo, Waterloo, Ontario, Canada; 4 School of Psychology and Sport Science, Bangor University, Bangor, Gwynedd, United Kingdom; 5 Schlegel-University of Waterloo Research Institute for Aging, University of Waterloo, Waterloo, Ontario, Canada; 6 Intuitive Surgical, Sunnyvale, California, United States of America; 7 School of Health and Huma Performance, Dalhousie University, Halifax, Nova Scotia, Canada; 8 School of Physical Therapy, University of Western Ontario, London, Ontario, Canada; 9 Cognitive Clinical Research Group, Parkwood Institute, London, Ontario, Canada; 10 Department of Medicine, Division of Cardiology, Schulich School of Medicine & Dentistry, University of Western Ontario, London, Ontario, Canada; Rutgers University Newark, UNITED STATES OF AMERICA

## Abstract

Monitoring middle cerebral artery blood velocity (MCAv) during maneuvers known to alter cerebral perfusion, such as supine-to-standing transitions or walking, may provide a more comprehensive assessment used to flag individuals susceptible to cerebral hypoperfusion in a way that cannot be achieved at rest. Furthermore, dual-tasks challenge the brain to match MCAv to meet increases in local demands of oxygen and energy in two different functional networks (motor and cognitive), potentially causing cerebral hypoperfusion when competing for shared and/or limited brain resources. We developed a dual-task paradigm comprising of five levels of task complexity, including single-tasks and dual-tasks. The main objective of the study was to evaluate changes in MCAv as task complexity increased, which was demonstrated through cognitive, motor, and combined cognitive-motor tasks in older adults with different cognitive function levels. A secondary objective was to assess the success rate (as a percentage) of obtaining MCAv signals during the dual-task protocol to determine the feasibility of measuring such metrics in older adults with varying levels of cognitive ability. Of the 88 participants (37 females, 75 ± 7 years, 27 ± 4 kg/m^2^), a MCAv signal was ascertained in 56 participants throughout both single-tasks and both dual-tasks. MCAv increased when transitioning from a simple single-task to a more complex dual-task, while also highlighting a decline in motor and cognitive performance. A full multi-modal signal acquisition (MCAv, blood pressure, and cerebral oxygenation) was acquired for 48 participants. Lower MCAv signal acquisition was observed in females and people with cognitive impairment. We have demonstrated how MCAv changes with increased task complexity, while also uncovering declines in gait and cognitive performance. By establishing the feasibility of obtaining MCAv signals during cognitive stress tests and dynamic movements in older adults with varying cognitive abilities, we can begin to assess cerebral hypoperfusion using a potentially more sensitive indicator linked to neural damage.

## Introduction

Worldwide, approximately 50 million people are living with dementia, with numbers projected to reach 75 million by 2030 and 132 million by 2050 [1,2]. Low cerebral blood flow is associated with cognitive impairment [3,4] and the development and progression of dementia [5–8]. During dynamic movements, such as walking, the cardiovascular and cerebrovascular systems are challenged by the redistribution of blood volume to the active muscles. Cerebral blood flow increases during moderate- to high-intensity exercise in response to altered arterial blood gases, increased blood pressure (BP) and cardiac output, neurogenic activity and changes in cerebral metabolism [9]. The higher brain regions, namely the central autonomic network (brain stem, cerebellum, and hypothalmus), are thought to regulate these changes in increased BP and cerebral blood flow during exercise [10].

When simultaneously walking and performing a cognitive task, known as dual-tasking, metabolic requirements are augmented in the different brain networks which are active for the required tasks; therefore, increasing demand on the cerebrovascular system’s capacity to redistribute blood flow to regions responsible for cognitive processing and motor performance. However, despite reductions in BP in the upright position compared to supine rest in older adults [11], few studies report middle cerebral artery blood velocity (MCAv) in older adults during activity due to reduced signal quality with increasing age [12,13] and movement artifacts or signal loss during activity. Importantly, assessing cerebral blood flow during dynamic movement can allow for a more comprehensive assessment of individuals with increased susceptibility to cerebral hypoperfusion (less than normal limits of cerebral blood flow [14]) in a way that cannot be achieved in a static supine position or during individual single-tasks.

The challenges of collecting MCAv during a dual-task paradigm with overground walking has limited previous researchers from assessing neurovascular coupling and dual-task cost simultaneously [15]. Portable transcranial Doppler ultrasound devices used to for MCAv signal acquisition have only been developed within the last decade, and previous cognitive stress tests used in a dual-task paradigm require either vocalization of the participant to communicate performance on the cognitive stress test (e.g., descending subtraction tasks), or visualization of the cognitive task (e.g., Stroop task). Therefore, the study objectives were to assess the changes of MCAv across five levels of task complexity, as demonstrated by cognitive tasks, motor tasks, and the combination of cognitive and motor tasks, and to determine the success rate (as a percentage) of acquiring MCAv signals in this dual-task protocol to identify the feasibility of testing such metrics in older adults with varying levels of cognitive function.

## Methods

Two hundred and six participants from the Comprehensive Assessment of Neurodegeneration and Dementia (COMPASS-ND) study were invited to partake in the current project from August 18^th^ 2016 for neuropsychological testing and dementia classifications used in this study, and recruitment of the 206 participants were completed on November 13^th^, 2021. Eighty-eight older adults (37:51 Female:Male; age: 75 ± 7 years) visited the Parkwood Institute in London, Ontario to participate in the current study and provided written, informed consent. The COMPASS-ND Study is a Canadian cohort research initiative, and it is a clinical study within the Canadian Consortium on Neurodegeneration in Aging (CCNA). All participants enrolled in the current sub-study (n = 88) were part of the CCNA and thus had previously been identified to have capacity to provide consent. All procedures were reviewed and approved by Clinical Trials Ontario (CTO750) at Western University and conformed with the Declaration of Helsinki. A full description of the COMPASS-ND informed consent, inclusion and exclusion criteria, and participant classification criteria has been published [16,17].

### Clinical group ascertainment

All participants were tested for cognitive capacity using the Montreal Cognitive Assessment (MoCA), in addition to standardized neuropsychometric testing and consensus diagnosis for categories of cognitive function. When assessing the feasibility of signal acquisition rates, participants were grouped as cognitively intact controls (CNT), having mild cognitive impairment (MCI) or having a clinical diagnosis of dementia (DEM).

Participants grouped as CNT (n = 22) had normal cognition or subjective cognitive impairment [18]. Briefly, to meet CNT group criteria, participants were required to have a Global Clinical Dementia Rating (CDR) equal to zero [19], verbal memory assessed with Logical Memory (2 above Alzheimer’s Disease Neuroimaging Initiative, ADNI, with education adjusted cut-offs) [20], Consortium to Establish a Registry for Alzheimer’s Disease (CERAD) [21], word list recall score >5 words (0–10 word scale) or Rey Auditory Verbal Learning-trial 7 (>6 words), and MoCA total score >24 [17,18].

Participant grouping as having MCI (n = 50) was assessed using the National Institute on Aging, Alzheimer’s Association Clinical Criteria (NIA-AA) [22]. MCI classification criteria included self-reported concerns of a change in cognitive function, impairment in one or more neuropsychological tests (*ADNI* Alzheimer’s Disease Neuroimaging initiative, CERAD, MoCA < 25, CDR 0.5), preserved independence (Lawton & Brody scale score >14), and absence of dementia based on CDR < 1 [17,22].

All classifications of dementia syndromes were made as per the COMPASS-ND protocol. Only those participants who were classified as Alzheimer’s dementia (n = 3), vascular cognitive impairment (n = 9), and mixed vascular and Alzheimer’s dementia (n = 4) were included in the dementia group (DEM n = 16) [17,16].

### Self-reported questionnaires

Upon arrival to the testing session, participants were asked to self-report the time since their last meal and/or beverage, which was on average 3 ± 3 hrs (median time of 2 hrs) prior to testing, and participants were asked the times and quantity of any caffeine intake during the previous 24 hrs. Participants were asked to complete five standardized questionnaires: Center for Epidemiological Studies Depression Scale [23], Orthostatic Hypotension Questionnaire [24], Composite Autonomic Symptom Score [25], Mini Nutritional Assessment [26], and the Orthostatic Discrimination and Severity Scale [27]. Participants were then asked to void their bladder before the equipment was set up.

### Hemodynamic testing

**Fig 1:**
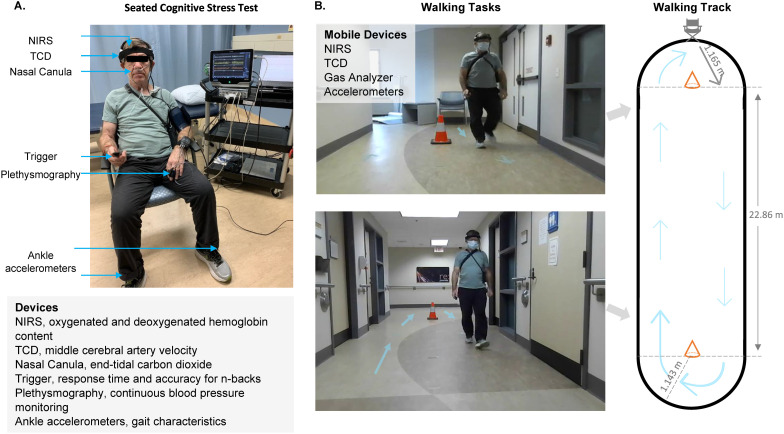
Equipment set up. A. Device set up, including a portable near infrared spectroscopy (NIRS) monitor to collect relative changes in oxygenated and deoxygenated hemoglobin content, portable transcranial Doppler ultrasound (TCD) device to monitor middle cerebral artery blood velocity, nasal cannula to collect end-tidal carbon dioxide, which was processed by a portable gas analyzer, Bluetooth connected trigger used to capture accuracy and response times on the cognitive stress test, plethysmography to collect continuous blood pressure, and two ankle accelerometers to record acceleration used for characterizing gait variability. B. Participant equipment set up for walking tasks and an illustration of the walking track at Parkwood Institute, London, Ontario.

The testing space was well-lit, and temperatures were held at 24 ± 1°C. Participants rested in the seated position and were instrumented with a portable transcranial Doppler ultrasound (TCD-X; Atys medical, Soucieu en Jarrest, France) device which was secured with a headset provided by the manufacturers, and cerebral tissue near-infrared spectroscopy (NIRS; Artinis Medical Systems BV, Netherlands) device which was secured with an elastic headband (Fig 1A). The middle cerebral artery runs laterally and slightly anteriorly; therefore, to insonate the M1 section of the artery the probe was positioned over the transtemporal window with a slight anterior tilt, allowing for a near-zero angle insonation, at a depth ranging from 35 to 55 mm, and with peak flow velocity tracings exported for analysis [28–30]. The middle cerebral artery was insonated using a 1.5-MHz transducer on the right side of the head, and if the signal quality was inadequate or not acquired, the left side was insonated (n = 14). It was deemed that the MCAv signal was inadequate if the outer envelope of the MCAv waveform was unattained or end-diastolic velocities were 0 cm/s or lower. During offline analysis (using Chart8: ADInstruments, Colorado, USA), the MCAv signal was filtered at 20 Hz and the mean waveform value was calculated for each beat to provide beat-to-beat analysis. The NIRS device was placed on the corresponding side of the TCD probe. Oxygenated (HbO_2_), deoxygenated (HHb), and total hemoglobin content (tHb) were measured as a change from baseline, and a calculated index of cerebral tissue oxygenation (tSO_2_) was obtained from the NIRS device manufacturers (NIRS; Artinis Medical Systems BV, Netherlands). The NIRS probe was placed over the right or left prefrontal lobe in accordance with the international 10–20 EEG landmarking system (right: Fp2, F4, F8, left: Fp1, F7, F3) [31]. The frontal lobe encompasses the prefrontal cortex which involves executive function, decision-making, working memory, and other higher-level cognitive functions. A source-detector distance of 4 cm was used for the HbO_2_, HHb, and tHb signals to reduce signal contamination from surrounding tissues [32]. The on-board quality control factor for the NIRS device indicated whether the tSO_2_ signal was adequate or not. A portable gas analyzer (Capnostream 35, Medtronics, USA and Ireland) estimated end-tidal carbon dioxide (ETCO_2_) from a nasal cannula where participants were instructed to only breathe through their nose. During non-walking tasks, arterial finger BP was monitored by a photoplethysmography (NOVA, Finapress Medical Systems, Amsterdam, Netherlands) device and adjusted to brachial BPs. All hemodynamic measures were averaged across 90 s to correspond with the single-task and dual-task blocks described below.

### Protocol design

#### Dual-task paradigm.

To assess each single-task independently as well as the effects of a dual-task, participants completed a working memory test in the seated position, a walking task, and two dual-tasks combining the working memory tests and walking (Fig 3) [33].

**Fig 2:**
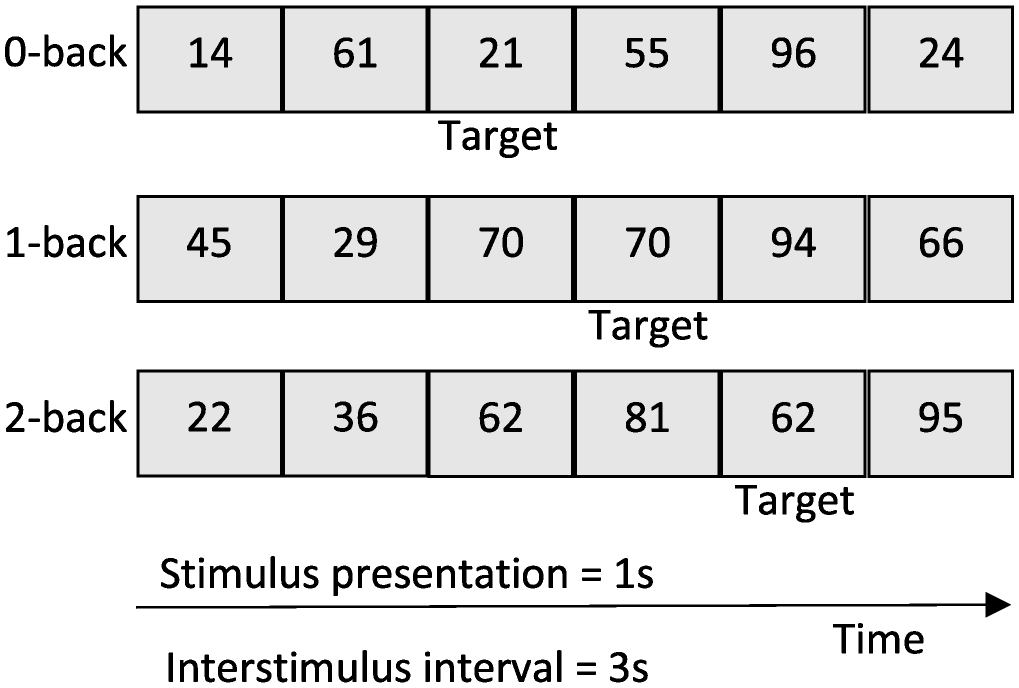
Depiction of the n-back protocol. The 0-back represented the low-cognitive-load conditions, and both the 1-back and 2-back represented the high-cognitive-load conditions.

#### Working memory performance.

Three auditory n-back conditions were developed: 0-back, 1-back, 2-back (Fig 2) [34,35]. Participants were presented with a sequence of stimulus and were instructed to respond when a target number (e.g., a number repetition, 1-back) was observed. Participants were instructed to press a button on a Bluetooth-connected clicker whenever they heard a target number. Each condition was 90 seconds in duration and presented either 6 targets (0-back) or 5 targets (1-back and 2-back). Each auditory stimulus was 1sec in duration. To select the target, participants were instructed to press the trigger on a Bluetooth-connected clicker (Fig 1A). Response times and target accuracy were collected on a standardized database. Participants were instructed to complete a practice session of the 0-back and 2-back conditions, and they were provided with feedback. If zero targets were identified in the 2-back practice session, participants completed the 1-back, rather than the 2-back condition, for an additional practice session and for all further testing. The seated 0-back (single-task-low-cognitive-load) and either the 1-back or 2-back (single-task-high-cognitive-load) were completed by all participants.

*Working memory protocol design:* During the single-task of working memory testing, participants sat quietly for 5 minutes before completing 4-blocks of n-back testing. Each block was a low- or high-cognitive-load condition, and each block was separated by 45 seconds of quiet rest. The block order for all participants was: low-, high-, low-, high-cognitive-load. During the dual-task, participants sat quietly for 5 minutes before walking and simultaneously completing either a low- or high-cognitive-load condition. Participants were asked to complete both dual-task conditions separately (dual-task-low-cognitive-load and the dual-task-high-cognitive-load), and they were completed in a random order.

*Behavioural performance on working memory performance test*: To differentiate between random target selection and information-based target detection, a performance-based signal detection variable was calculated as Sensitivity A’.


$$\begin{array}{c} \begin{array}{c} \text{Sensitivity A}’=\text{0}.\text{5}+((\text{hit rate}-\text{false alarm rate})\\ \times (\text{1}+\text{hit rate}-\text{false alarm rate}))/(\text{4}\times \text{hit rate}\times (\text{1}-\text{false alarm rate})) \end{array} \end{array}$$

[35–37], where a value of 1 equates to the perfect discernment between targets and non-target stimuli, and a value of 0.5 represents random target selection. To account for the speed versus accuracy exchange, a sensitivity A’ score was calculated.


$$\text{Sensitivity}{\text{A}}^{\prime }\text{score}=\text{100 x sensitivity A}’/\text{reaction time on targets}(\text{ms})\text{x 100}$$

[38,39]. The Sensitivity A’ score was calculated for each 90 s block.

#### Motor task performance.

Walking is a frequently performed activity in daily life and was selected as the motor task. Sixteen of the participants used assistive devices for the walking task (walker n = 7, cane n = 7, other n = 2). Participants were instrumented with a tri-axial accelerometer (GENEactive; Activinsights, Kimbolton, UK) on the lower lateral shank of each ankle with a sample rate set to 50 Hz. Three walking conditions were completed in a randomized order, single-task-walking (WALK), dual-task-low-cognitive-load, and dual-task-high-cognitive-load. All walking conditions were preceded by 5 minutes of seated rest, followed by 90 s of walking. Participants were instructed to walk at a self-selected usual pace. Walking time was defined as the time from the first toe-off to the final heel strike at 90 s and the gait speed was calculated as the distance traveled divided by 90 s. The walking track was comprised of 2 semicircles, each with a perimeter walking path of 1.8 meters, which were joined by two straight paths, each 22.86 meters in length (Fig 1B).

### Statistical analysis

Data were analyzed using SPSS software (version 26, IBM Corp, Armonk, NY, 2019). The MCAv, gait speed, and cognitive performance data were checked for normality (a Kurtosis values less than three, Kolmogorov-Smirnova with a p-value greater than 0.05) as well as homogeneity of variance (Mauchly’s test of sphericity with a p-value greater than 0.05, if p ≤ 0.05 then the Greenhouse-Geisser p-value was used). A one-way repeated measures ANOVA was performed to determine differences among motor performance conditions which included all overground walking at a self-selected pace (WALK, dual-task-low-cognitive-load, and dual-task-high-cognitive-load). A one-way repeated measures ANOVA was also performed to determine differences among cognitive performance condition all of which included n-back testing (single-task-low-cognitive-load, dual-task-low-cognitive-load, single-task-high-cognitive-load, dual-task-high-cognitive-load). A Bonferroni correction factor was applied, and pairwise comparisons were performed to identify differences between conditions. If the data were not normally distributed a Friedman test was applied, followed by Wilcoxon signed ranks test to identify between condition differences. Significance was defined as a p-value equal to or less than 0.05.

## Results

### Participant characteristics at intake

**Fig 3:**
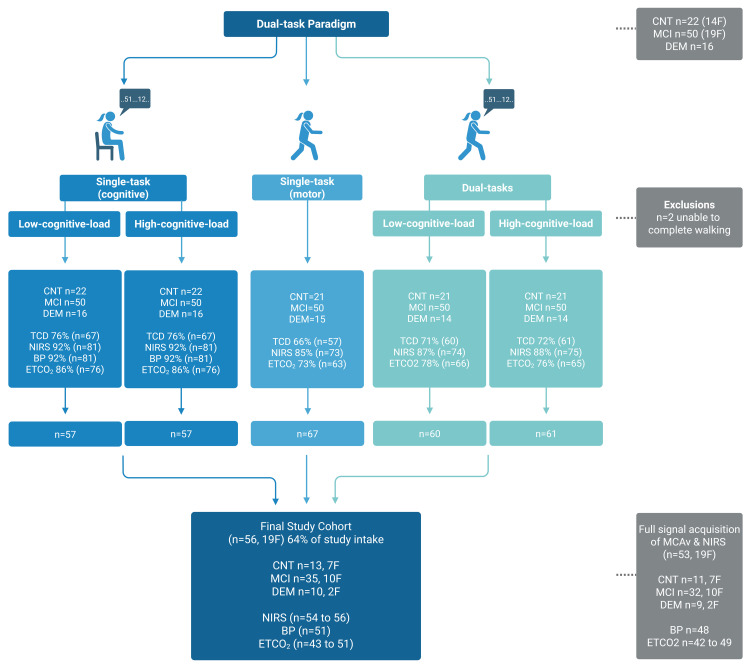
Design and flow chart of study. Study breakdown of vascular measure for each protocol component. *F* females, *CNT* cognitively intact controls, *MCI* mild cognitive impairment, *DEM* dementia, *NIRS* portable near infrared spectroscopy monitor to collect relative changes in oxygenated and deoxygenated hemoglobin content, *TCD* portable transcranial Doppler ultrasound device to monitor middle cerebral artery velocity, *ETCO*_*2*_ end-tidal carbon dioxide, *BP* continuous monitoring of beat-to-beat blood pressure, *DT* dual-task.

Eighty-eight participants arrived for motor, cognitive, and hemodynamic testing (CNT n = 22, MCI n = 50, DEM n = 16) where 38 of these participants self-identified as female at birth (Table 1, Fig 3). Of the 88 participants at intake, participants were 75 ± 7 years old, had a body mass index of 27 ± 4 kg/m^2^, had 16 ± 3 years of formal education and only 6 were left-handed (Table 1). Thirty-two participants were excluded from the final study cohort as they were unable to complete the walking tasks (n = 2), or had an inadequate or absent MCAv signal (described in detail below). The remaining 56 participants comprised the final study cohort (Fig 3, Table 1) and had a mean age of 75 ± 7 years old, a body mass index of 26 ± 4 kg/m^2^, had 16 ± 4 years of formal education, and only 4 were left-handed (Table 1). The data distribution characteristics (e.g., normality, skewness, kurtosis) of the final study cohort are presented in S1 File and S2–S6 Tables.

**Table 1:** Participant Characteristics.

	Study intake	Final study cohort
Sample size, n	88	56
Cognitively intact controls, n (%)	22 (25%)	13 (23%)
Mild cognitive impairment, n (%)	50 (57%)	35 (63%)
Dementia, n (%)	16 (18%)	10 (18%)
Female, n (%)	38 (43%)	19 (34%)
Age, years, avg ± sd	75 ± 7	75 ± 7
BMI, kg/m^2^, avg ± sd	27 ± 4	26 ± 4
Education, years, avg ± sd	16.0 ± 3.4	16.2 ± 3.6
MoCA score out of 30, avg ± sd	23.6 ± 4.9	23.4 ± 5.3
Left-handed, n (%)	6 (7%)	4 (7%)
Use of an assistive device, n (%)	16 (18%)	9 (16%)
Walker, n (%)	7 (8%)	2 (4%)
Cane, n (%)	7 (8%)	5 (9%)
Other, n (%)	1 (1%)	1 (2%)
**Cardiovascular Characteristics**		
Systolic BP (mmHg), avg ± sd	132 ± 15	133 ± 14
Diastolic BP (mmHg), avg ± sd	74 ± 8	74 ± 8
On BP lowering meds, n (%)	33 (38%)	21 (38%)
TCD collected on the left side, n (%)	14 (16%)	9 (16%)

*avg* average, *sd* standard deviation, *BMI* body mass index, *MoCA* Montreal Cognitive Assessment, *BP* blood pressure, *meds* medications, *ARBs* Angiotensin receptor blockers, *TCD* transcranial Doppler ultrasound.

### Cerebral blood velocity and task complexity of the final study cohort

**Fig 4:**
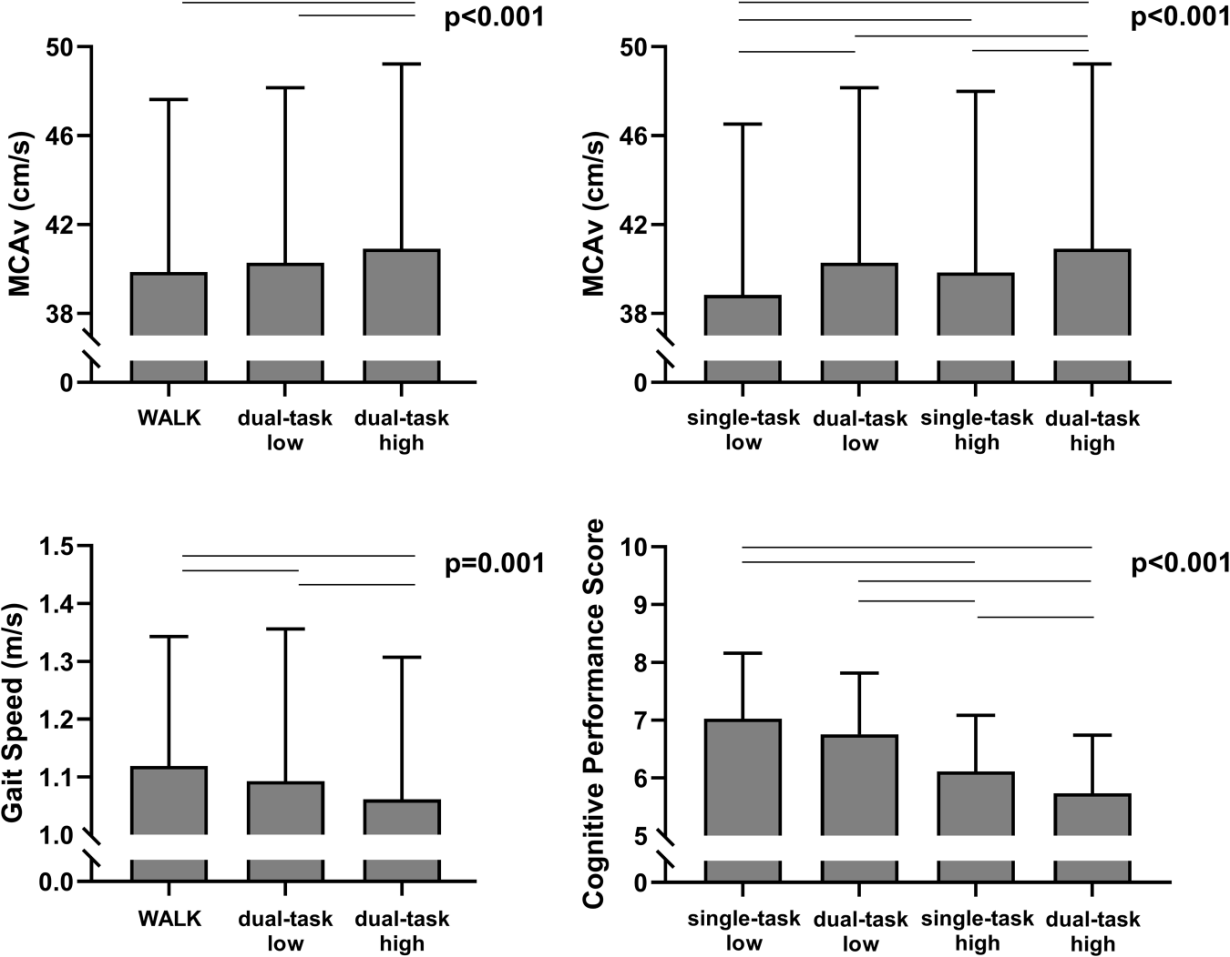
Middle cerebral artery blood velocity (MCAv) during three motor tasks with increasing task complexity (left panel), and during four cognitive tasks with increasing task complexity (right panel). Repeated measures ANOVA results were p < 0.01 for all comparisons and significant post-hoc differences are p < 0.05 (solid lines), and non-significant trends are p < 0.1 and p > 0.05 (dashed lines). All motor tasks were completed at a self-selected pace during overground walking. Conditions: single-task walking (WALK), single-task-low-cognitive-load, dual-task-low-cognitive-load, single-task-high-cognitive-load, and dual-task-high-cognitive-load.

A repeated measures ANOVA was conducted to examine the effect of different conditions (walk, dual-task-low-cognitive-load, dual-task-high-cognitive-load) on MCAv. The analysis revealed a significant main effect of condition, F(2, 54)=10.601, p < 0.001, η^2^p=0.162. This indicates that the condition had a significant impact on MCAv, with the partial eta-squared value indicating a large effect size. Post-hoc tests with a Bonferroni correction identified that the dual-task-high-cognitive-load led to significantly greater MCAv compared to the walk (p < 0.001) and the dual-task-low-cognitive-load (p = 0.02, Fig 4). Among the three conditions which included overground walking at a self-selected pace (WALK, dual-task-low-cognitive-load, and dual-task-high-cognitive-load), as the task complexity increased MCAv also increased (p < 0.001, Fig 4), whereby the dual-task with the more difficult cognitive stress test (dual-task-high-cognitive-load) had a significantly greater MCAv compared to the single-task of walking (WALK), and the simple dual-task (dual-task-low-cognitive-load). Non-parametric Friedman’s test results identified gait speed decreased with increased task complexity (p = 0.001) where the Wilcoxon signed ranks test for gait speed identified that the walk was faster than the dual-task-low-cognitive-load (p = 0.03), and the dual-task-low-cogntive-load was faster than the dual-task-high-cognitive-load (p = 0.032, Fig 4 and S5 Table). ETCO_2_ was not significantly different between conditions.

A repeated measures ANOVA was conducted to examine the effect of different conditions (single-task-low-cognitive-load, single-task-high-cognitive-load, dual-task-low-cognitive-load, dual-task-high-cognitive-load) on MCAv. The analysis revealed a significant main effect of condition, F(2, 54)=12.281, p < 0.001, η^2^p=0.183. This indicates that the condition had a significant impact on MCAv, with the partial eta-squared value indicating a large effect size. Post-hoc tests with a Bonferroni correction identified that the single-task-low-cognitive-load was significantly lower than the single-task-high-cognitive-load (p < 0.001), dual-task-low-cognitive-load (p = 0.005), and the dual-task-high-cognitive-load (p < 0.001, Fig 4). Post-hoc tests with a Bonferroni correction also identified that the single-task-high-cognitive-load is significantly lower than the dual-task-high-cognitive-load (p = 0.047), and the dual-task-low-cognitive-load is significantly lower than the dual-task-high-cognitive-load (p = 0.04, Fig 4). When comparing the four conditions involving the cognitive stress test (single-task-low-cognitive-load, dual-task-low-cognitive-load, single-task-high-cognitive-load, dual-task-high-cognitive-load), MCAv significantly increased as the task complexity and cognitive load increased (p < 0.001, Fig 4). Pairwise comparisons identified significant differences of MCAv among all conditions with the exception of single-task-high-cognitive-load and dual-task-low-cognitive-load (Fig 4). Non-parametric Friedman’s test results identified cognitive performance scores significantly decreased with increasing task complexity (p < 0.001, Fig 4). Wilcoxon signed ranks test revealed that cognitive performance was reduced between all conditions with the exception of single-task-low-cognitive-load and dual-task-low-cognitive load (S4 Table). Importantly, cognitive performance scores were reduced from the single-task-low-cognitive-load to the single-task-high-cognitive-load (p < 0.001), from the single-task-high-cognitive-load to the dual-task-low-cognitive-load (<0.001), from the dual-task-low-cognitive-load to the dual-task-high-cognitive-load (p < 0.001, Fig 4). ETCO_2_ was not significantly different between conditions.

### Final study cohort – Breakdown of MCAv signal acquisition by clinical group and sex

**Fig 5:**
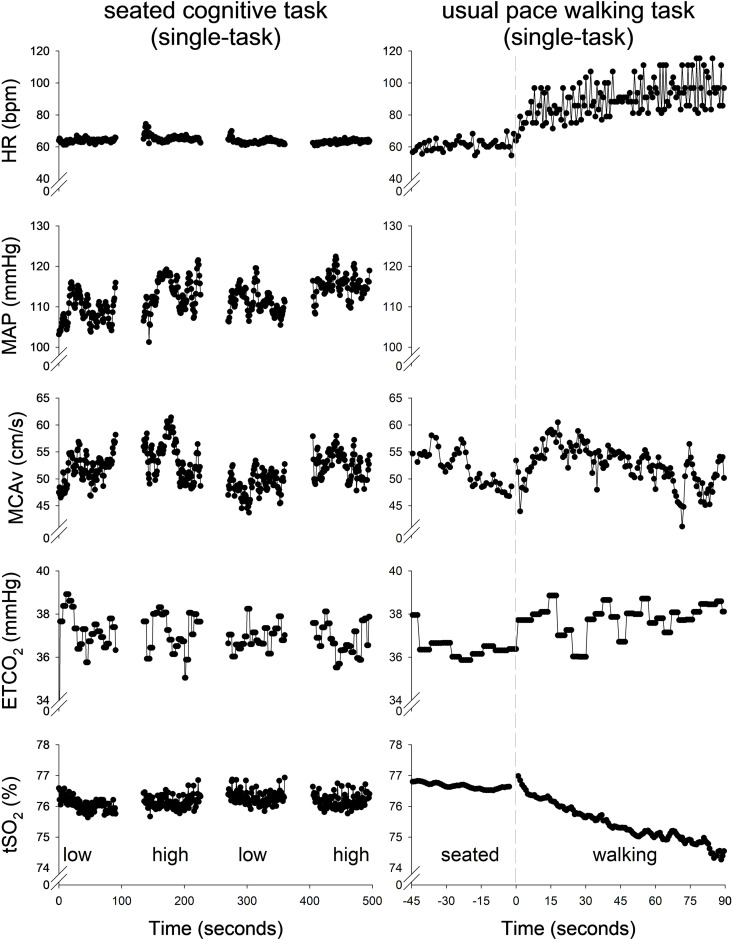
Representative data for both types of single-tasks. Representative data (n = 1) during the seated cognitive tasks (left panel) and the usual pace walking task (right panel) for heart rate (HR), mean arterial pressure (MAP) middle cerebral artery velocity (MCAv), end-tidal carbon dioxide (ETCO_2_), and cerebral tissue saturation (tSO_2_). MAP during overground walking (left panel) could not be assessed without a portable continuous blood pressure device. The seated cognitive tasks follow a 90 s block design in the order of control-task-control-task, with 45 seconds between blocks. The usual pace walking task began with seated rest (negative time), followed by a 90-s walking task, where time at zero marks the first heel strike of walking.

Of the 56 participants in the final study cohort, 19 (34%) were female. Within each clinical group, there were 7 (54%) female CNT participants, 10 (29%) female MCI participants, and 2 (20%) female DEM participants (Table 2, Fig 3). The MCAv signal and the individual time course of HR, continuous BP, ETCO_2_, and tSO_2_ during the seated cognitive stress test and walking task (both single-tasks) are demonstrated in Fig 5. Signal acquisition rates of tSO_2_ ranged from 85% to 88% during overground walking conditions, was 92% during seated conditions, and had a 96% to 100% success rate for the final study cohort. Of the 88 participants at study intake, 13 (59%) of CNT participants, 35 (70%) of MCI participants, and 10 (63%) of DEM participants completed all protocol components with an adequate MCAv signal (Fig 3). Of the 88 participants at study intake, 48 remained who had multi-modal data acquisition of MCAv, NIRS and BP for all conditions, which included 11 (23%) CNT participants, 28 (58%) MCI participants, and 9 (19%) DEM participants.

**Table 2:** Group by sex sample size breakdown.

Groups	Study intake			Final study cohort			Retained from study intake to final cohort		
	F	M	C	F	M	C	F	M	C
Overall, n	38	50	88	19	37	56	50%	74%	64%
CNT, n	14	8	22	7	6	13	50%	75%	59%
MCI, n	19	31	50	10	24	34	53%	77%	68%
DEM, n	5	11	16	2	7	9	40%	64%	56%

*CNT* cognitively intact controls, *MCI* mild cognitive impairment, *DEM* dementia, *F* females, *M* males, *C* combined females and males, *% Retained* percentage of participants in final study cohort compared to study intake for F, M, and C respectively.

## Discussion

In this cross-sectional observational study, we investigated the relationship of MCAv during incremental increases of task complexity while using portable devices in a cohort of older adults with varying levels of cognitive ability. As task complexity increased, MCAv also significantly increased whereas the performance metrics of gait speed and cognitive performance scores decreased. We also examined the feasibility of measuring MCAv during a dual-task paradigm, which included two single-tasks and two dual-tasks. The target population for this study was older adults with varying levels of cognitive function (CNT, MCI, and DEM). To judge the feasibility of the study, MCAv signal acquisition across all protocol components was required. Of the 88 participants at the study intake, 56 participants remained in the final study cohort. A lower MCAv success rate was observed in females (Fig 3, Table 2) and, the DEM group consistently had the lowest MCAv signal acquisition rate (Fig 3, Table 2).

### MCAv and task complexity

This is the first study to demonstrate an increase in MCAv from a simple to a more complex dual-task while simultaneously demonstrating reduced motor task performance. Previous researchers have utilized treadmills [40], which do not precisely measure the dual-task cost of a slower gait speed. Furthermore, previous researchers have asynchronously assessed MCAv and cognitive performance on an n-back with a separate motor task [41], but not simultaneously to assess neurovascular coupling alongside dual-task cost. During overground walking we observed MCAv to significantly increase during the difficult dual-task (dual-task-high-cognitive-load) compared to both of the other walking conditions (p < 0.001, Fig 4) without any obvious differences in ETCO_2_ to drive the changes in MCAv. The potential role of increases in mean arterial pressure during walking could not be assessed without a portable continuous BP device. Gait speed had a concomitant but opposite response to MCAv where gait speed decreased with the more complex dual-task (dual-task-high-cognitive-load) compared to the simple dual-task (dual-task-low-cognitive-load) and the single-task of walking (WALK, p = 0.001, Fig 4). During the cognitive tasks, MCAv incrementally increased with greater task difficulty (<0.001, Fig 4), where post-hoc analysis identified differences between all conditions with the exception of a difference between dual-task-low-cognitive-load to single-task-high-cognitive-load. Similarly, cognitive performance scores decreased as the task complexity increased (p < 0.001). It has previously been established that there are age-related reductions in neurovascular coupling using n-back tasks to assess working memory with NIRS [42] and with dementia [43] but this is the first evidence within a cohort of older adults with varying levels of cognitive function that a hyperemic response of MCAv is coupled with reduced motor task performance and cognitive performance metrics when collected simultaneously.

### MCAv feasibility

This was a proof-of-concept study in participants with varying levels of cognitive function, where the primary aim of the trial was to determine if alterations in MCAv during dynamic movement and dual-tasks are a feasible measure for assessment in this population. We utilized validated measures of MCAv as an indicator of altered cerebral hemodynamics in older adult participants.

Dynamic activity, such as walking or a repeated sit-to-stand maneuver, can reduce the protocol sample size due to physical limitations of the protocol and add motion artifacts to the MCAv signal, rendering an inadequate MCAv quality. The current study had an attrition rate of 2–3% between the seated cognitive stress test and the ability of participants to complete the walking tasks. Ascertaining a stable and successful MCAv signal during the static seated cognitive stress test yielded only slightly higher MCAv signal success (76%) compared to the three conditions involving dynamic movement (66% to 72%). The signal acquisition rates between static and dynamic protocols suggest vessel insonation was the primary barrier to lower MCAv data attainment and not the motor task (Fig 3). Attaining a high-quality MCAv signal is reliant on the penetration of the ultrasound beam through the transtemporal acoustic window. The transtemporal acoustic window is negatively affected by increased transtemporal bone thickness, reduced bone density, inhomogeneous bone composition, age, female sex, and non-Caucasian ethnicity [13,44–46]. The age-related changes in transtemporal bone composition in females are thought to coincide with characteristic changes observed in bone with menopause [44,45]. These changes in bone composition result in greater scattering and absorption of the ultrasound beam, reducing the passage of the signal through the tissue and resulting in a poor quality or absent signal [44]. Older Japanese adults who are 70 + years and female have a bilateral MCAv success rate of 17% [12]. In comparison, young male and female adults can have a 100% success rate [47]. Vessel tortuosity increases with age [48] which could contribute to difficulty in ascertaining a MCAv signal, and increased brain atrophy with age [49] may also present challenges sampling deep enough within the brain to attain a MCAv signal.The present study observed 50% of females and 74% of males at study intake had an adequate MCAv signal throughout the study protocols (Table 2).

In both the females and males, the DEM group had the lowest percentage of MCAv signal acquisition (Fig 3, Table 2). The current observations suggest females and participants with dementia have a lower success rate of acquiring a MCAv signal and physically being able to complete the static and dynamic testing involved in the proposed dual-task paradigm. Previous reports demonstrate a lower combined MCAv and continuous BP signal success rate in older adults with DEM compared to CNT or MCI during rest (81%−85% in CNT, 76%−83% in MCI, and 67%−76% in DEM) and during a repeated sit-to-stand maneuver (86%−88% in CNT, 86% in MCI and 74% in DEM) [50]. The marginally higher MCAv failure rates of the current investigation could be attributed to the required use of everyday glasses during the walking tasks. Participants with either large glasses frames/temples or temples which directly obstructed the transtemporal acoustic window rendered vessel insonation inaccessible. The unrecorded number of participants wearing glasses, which obstructed the transtemporal window, is a study limitation.

### NIRS, BP and ETCO_2_ device feasibility

The current study observed tSO_2_ NIRS signals for 85%−92% of participants, which is lower than typically observed in older or younger adults (Fig 3) [47,51]. A failure to attain the NIRS signal was attributed to poor device-to-skin contact due to deep forehead wrinkles (n = 2), signal-device crosstalk during an experimental application of dual-NIRS application on the left and right prefrontal cortex (n = 3), technician set-up error (n = 3), movement artifacts and light exposure during walking (n = 7). Some of these causes for the low signal attainment (e.g., experimental dual-NIRS application on the left and right prefrontal cortex, or light exposure during walking) could be avoided in future data collections. Despite the unusually low signal acquisition rate for the NIRS device, a greater success rate for data collection was acquired with NIRS compared to TCD (MCAv).

Continuous BP measures were collected during the cognitive stress test in 92% of participants. The inability to acquire a BP plethysmography signal in 7 participants was attributed to low peripheral blood flow (cold hands) and high systolic BPs.

Successful data collection for ETCO_2_ ranged from 73%−86% (Fig 3). Equipment set-up of the nasal cannula (Fig 1) and initial data collection was completed on 100% of participants; however, due to strict COVID-19 regulations during the data collection in 2021, equipment limitations (unable to reuse nafion tubing between participants or apply a 3-way valve to the open air) resulted in the accumulation of moisture in the nasal cannula lines and excessively low data acquisition rates.

Full signal acquisition of MCAv, NIRS, and BP across all single- and dual-task conditions was 60% (n = 53/n = 88, Fig 3). Previous work in cognitively impaired older adults has reported a much lower combined signal success rate for MCAv, NIRS, and BP to be 37% [52]. The current investigation can report 48 (55%) of participants from study intake had combined MCAv, NIRS, and BP data throughout all study components, and of these 48 participants, 17 (35%) were female, and only 9 (19%) were from the DEM group. This finding is important for physiological research as older adults, particularly females and those with cognitive impairment are often under-represented in research, yet may be the most likely to benefit from treatments derived from the research. Our study supports the feasibility of multi-modal, cardiovascular and cerebrovascular research in older adults with cognitive impairment.

## Conclusions

Across five levels of task complexity, we demonstrated that MCAv increases with rising task demands, while simultaneously demonstrating compromised performance on gait and cognitive metrics. The equipment selected for this protocol was portable and unencumbering, which combined with the innovative approach to delivering a working memory task, we accurately and precisely ascertained an MCAv signal, gait metrics, and cognitive performance in 66% of older adults with diverse levels of cognitive function during dual-tasks. The current findings suggest that the proposed dual-task paradigm is well tolerated, and signal acquisition is feasible in this population.

Many older adults and their families suffer from the effects of dementia worldwide [53–56], which may have vascular underpinnings [3–8]. The goal of this dual-task paradigm with overground walking was to provide evidence for the feasibility to link the relationships between MCAv with neural function during dynamic activities of every day living. We have found that TCD is a viable way to capture dynamic changes in MCAv with changes in cognitive load, and we expect our findings will hold the potential to shape early detection assessments techniques for the development and progression of cognitive impairment.

## Supporting information

S1 File Data Distribution Results. (PDF)

S2 Table Data Distribution Test Results. (PDF)

S3 Table Friedmans Test Results. (PDF)

S4 Table Wilcoxon Signed Ranks Test for Cognitive Performance Score. (PDF)

S5 Table Wilcoxon Signed Ranks Test for Gait Speed. (PDF)

S6 Table Mauchly’s Test of Sphericity, Effect Size and Power for Each Model Comparison. (PDF)
