# Supplementary material for: Navigating the brain: How cerebral blood flow shifts with task complexity
Source: PLoS One. 2025 Oct 23;20(10):e0333684. doi: 10.1371/journal.pone.0333684 (PMC12548881; doi:10.1371/journal.pone.0333684)
Supplement: S1 File — (PDF) [file pone.0333684.s001.pdf]

S1 File. Data Distribution Results

When examining the distribution of the data, all of the MCAv metrics were normally distributed, were not skewed, and did not have kurtosis. However, the dual-task low condition for cognitive performance had a Kolmogorov-Smirnov result suggesting a non-normal distribution ($p=0.035$, Table S2) and the single-task low condition for cognitive performance had a kurtosis value greater than 3 (value=3.403) suggesting a sharper peak and heavier tails compared to a traditional normal distribution curve (Table S2). A Friedman Test and Wilcoxon Signed Ranks Test were subsequently conducted (Table S3-S5). Gait speed was not skewed and did not have kurtosis, but it was abnormally distributed (Table S2). The Friedman's test suggests a significant difference between conditions for gait speed and the Wilcoxon signed ranks test identifies differences between all conditions (Table S3 and Table S5).

A Mauchly's test of sphericity was performed to investigate the homogeneity of variance for MCAv comparing the single-task low, single-task high, dual-task low, and dual-task high conditions which yielded a $p<0.001$, therefore the Greenhouse-Geisser p-value ($p=0.000$) has been reported (Table S6). A Mauchly's test of sphericity was performed on the MCAv motor tasks, and cognitive performance scores (Table S6) whereby the Sphericity assumed p-values were reported in the manuscript results. A Friedman's test was performed on the cognitive performance scores and gait speed comparisons and a Wilcoxon signed ranks identified differences between conditions (Table S5).
