# Supplementary material for: Navigating the brain: How cerebral blood flow shifts with task complexity
Source: PLoS One. 2025 Oct 23;20(10):e0333684. doi: 10.1371/journal.pone.0333684 (PMC12548881; doi:10.1371/journal.pone.0333684)
Supplement: S2 Table — (PDF) [file pone.0333684.s002.pdf]

Table S2. Data Distribution Test Results

Condition	Skewness	Kurtosis	Kolmogorov-Smirnov
**MCAv during cognitive performance**			
Single-task low	-0.076	-0.213	0.200
Single-task high	-0.037	-0.270	0.200
Dual-task low	0.353	0.341	0.056
Dual-task high	0.269	0.062	0.200
**MCAv during motor tasks**			
Walk	0.386	0.213	0.186
Dual-task low	0.353	0.341	0.056
Dual-task high	0.269	0.062	0.200
**Cognitive Performance**			
Single-task low	-0.973	3.403*	0.165
Single-task high	-0.434	-0.102	0. 200
Dual-task low	-0.950	0.202	0. 035*
Dual-task high	-0.354	0.223	0.200
**Gait Speed**			
Walk	0.093	0.054	0. 050*
Dual-task low	-0.101	0.305	0.031*
Dual-task high	-0.004	-0.021	0.200

* Suggests non-normality, *MCAv* middle cerebral artery blood velocity
