# Supplementary material for: Navigating the brain: How cerebral blood flow shifts with task complexity
Source: PLoS One. 2025 Oct 23;20(10):e0333684. doi: 10.1371/journal.pone.0333684 (PMC12548881; doi:10.1371/journal.pone.0333684)
Supplement: S4 Table — (PDF) [file pone.0333684.s004.pdf]

Table S4. Wilcoxon Signed Ranks Test for Cognitive Performance Score

	Single-task low VS single-task high	Single-task low VS dual-task low	Single-task low VS dual-task high	Single-task high VS dual-task low	Single-task high VS dual-task high	Dual-task low VS Dual-task high
Z	-5.591b	-1.434b	-5.901b	-4.267c	-2.186b	-4.732b
p-value	0.000	0.152	0.000	0.000	0.029	0.000

b based on positive ranks, c based on negative ranks
