# Supplementary material for: Navigating the brain: How cerebral blood flow shifts with task complexity
Source: PLoS One. 2025 Oct 23;20(10):e0333684. doi: 10.1371/journal.pone.0333684 (PMC12548881; doi:10.1371/journal.pone.0333684)
Supplement: S5 Table — (PDF) [file pone.0333684.s005.pdf]

Table S5. Wilcoxon Signed Ranks Test for Gait Speed

	Walk VS dual-task low	Walk VS dual-task high	Dual-task low VS Dual-task high
Z	-2.170b	-3.630b	-2.141b
p-value	0.030	0.000	0.032

b based on positive ranks
