# Supplementary material for: Navigating the brain: How cerebral blood flow shifts with task complexity
Source: PLoS One. 2025 Oct 23;20(10):e0333684. doi: 10.1371/journal.pone.0333684 (PMC12548881; doi:10.1371/journal.pone.0333684)
Supplement: S6 Table — (PDF) [file pone.0333684.s006.pdf]

Table S6. Mauchly's Test of Sphericity, Effect Size and Power for Each Model Comparison

Condition and variables	Mauchly's test of Sphericity	Sphericity assumed p-value	Greenhouse-Geisser p-value	F Score	Partial Eta Squared	Observed Power
**MCAv during cognitive tasks**	<0.001	<0.001	<0.001	12.281	0.183	0.973
Single-task low						
Single-task high						
Dual-task low						
Dual-task high						
**MCAv during motor tasks**	0.143	<0.001	N/A	10.601	0.162	0.988
Walk						
Dual-task low						
Dual-task high						
**Cognitive Performance**	0.069	<0.001	N/A	32.748	0.396	1.000
Single-task low						
Single-task high						
Dual-task low						
Dual-task high						
**Gait Speed**	0.123	0.001	N/A	8.126	0.129	0.955
Walk						
Dual-task low						
Dual-task high						

Partial Eta Squared (η^2) is a measure of effect size and the observed power was computed using alpha 0.05 for each model. NA not applicable because the Maulchy's test of Sphericity had a p-value greater than 0.05. *MCAv* middle cerebral artery blood velocity
